# Effect of disinfecting the cavity with chlorhexidine on the marginal gaps of Cl V giomer restorations

**DOI:** 10.4317/jced.53193

**Published:** 2017-02-01

**Authors:** Soodabeh Kimyai, Fatemeh Pournaghi-Azar, Fereshteh Naser-Alavi, Ashkan Salari

**Affiliations:** 1 Dental and Periodontal Research Center, Faculty of Dentistry, Tabriz University of Medical Sciences, Tabriz, Iran; 2 Professor, Department of Operative Dentistry, Faculty of Dentistry, Tabriz University of Medical Sciences, Tabriz, Iran; 3 Assistant Professor, Department of Operative Dentistry, Faculty of Dentistry, Tabriz University of Medical Sciences, Tabriz, Iran; 4 Post graduate student, Department of Operative Dentistry, Faculty of Dentistry, Tabriz University of Medical Sciences, Tabriz, Iran; 5 Post graduate student, Department of Periodontics, Faculty of Dentistry, Tabriz University of Medical Sciences, Tabriz, Iran

## Abstract

**Background:**

Considering the effect of cavity disinfecting agents on the bonding and sealing ability of restorations bonded to dentin, the aim of this study was to evaluate the effect of chlorhexidine (CHX) disinfecting agent on the marginal gaps of Cl V giomer restorations.

**Material and Methods:**

Cl V cavities were prepared on the buccal surfaces of 60 sound bovine permanent incisors in this *in vitro* study, with the occlusal and gingival margins in enamel and dentin, respectively. The teeth were randomly divided into two groups (n=30). The teeth in groups 1 and 2 were restored without and with the use of the disinfecting agent in the cavity, respectively, before applying the adhesive. BeautiBond one-step self-etch adhesive and Beautifil II giomer were used to restore the cavities in both groups. After thermocycling and sectioning of the samples, the sizes of marginal gaps at gingival margins were measured in µm under a stereomicroscope. Mann-Whitney U test was used to compare marginal gaps at *P*<0.05 level of significance.

**Results:**

The means of marginal gaps were significantly different between the two study groups (U=180, *P*<0.001), with higher means of marginal gaps in group 2 (with CHX disinfection) compared to group 1 (without CHX disinfection) (*P*<0.0005).

**Conclusions:**

Application of CHX for the disinfection of cavities in giomer restorations resulted in an increase in gingival margin gaps.

** Key words:**Chlorhexidine, dental marginal adaptation, dental restorations.

## Introduction

Currently, use of cavity disinfecting agents has become popular after preparing the cavity and before placing the restorative material (1). During the tooth preparation stage, it is not possible to completely eliminate bacteria from the cavity even with the use of disclosing dyes and it has been reported that the bacteria remaining in the cavity can preserve their activity for some time in the dentin (for more than a year) (2,3). It appears there is greater need for disinfection of the cavity in the self-etch bonding systems due to the absence of the irrigation step and removal of the smear layer (4). Various antibacterial agents, such as chlorhexidine (CHX), sodium hypochlorite, fluoride-based solutions and benzalkonium chloride can be used as disinfecting agents in the cavity (5,6). CHX has been suggested as an effective agent for the disinfection of cavity (7). CHX is a water-soluble material and can inhibit bacteria by bonding to Ca2+ sites at physiologic pH levels (8).

However, some researchers believe that use of disinfecting agents can affect the sealing ability and seal of restorations bonded to dentin and result in an increase in microleakage (1,9). The presence of microleakage and its persistence gives rise to tooth sensitivity, margin discoloration, recurrent caries and irritation of the pulp (10). Previous studies have evaluated the effect of cavity disinfection on the bonding of composite resin restorations and have reported different results based on the type of the disinfecting agent and the type of the bonding system used (1,9,11). Türkün *et al.* reported that use of CHX and benzalkonium chloride had no effect on the enamel and dentin margin microleakage of composite resin restorations bonded with self-etch adhesives, but the use of iodine compounds resulted in a significant increase in microleakage (1). However, Tulunoglu *et al.* reported that CHX had a negative effect on the sealing ability of composite resin restorations bonded with one-bottle total-etch adhesives at gingival margins (9). Hiraishi *et al.* reported an increase in microleakage and a decrease in the microtensile bond strength of composite resin blocks cemented with resin cements in associations with a self-etch adhesive subsequent to the use of CHX disinfecting agent (11). In contrast, in another studies application of CHX did not cause loss of dentin bond strength of adhesives (12-14).

In recent years, a new generation of bonded materials, referred to as giomers, have been introduced for direct restorations. Glass-ionomer fillers have been incorporated into the resin matrix of these light-cured materials. These materials have the advantages of composite resins (superb esthetics, easy polishability and biocompatibility) and glass-ionomers (fluoride release and fluoride recharge) at the same time (15).

Since no studies to date have evaluated the effect of cavity disinfectants on the marginal gaps of giomer restorations, the aim of this *in vitro* study was to evaluate the effect of disinfecting the cavity with CHX on the marginal gaps of Cl V restorations with the use of a one-step self-etch adhesive.

## Material and Methods

The study protocol was approved by the Ethics Committee at Tabriz University of Medical Sciences. Sixty sound bovine permanent mandibular incisors were included in this *in vitro* study.

Sample size determination: We planned a study of a continuous response variable from independent control and experimental subjects with one control per experimental subject. In a pilot study the response within each subject group was normally distributed with the standard deviation of 3.5. If the true difference in the experimental and control means was 4, we would need to study 26 experimental subjects and 26 control subjects to be able to reject the null hypothesis that the population means of the experimental and control groups were equal with the power of 0.8. The Type I error probability associated with the test of this null hypothesis was 0.5. In order to increase the validity, the sample size was considered 30 in each group.

The inclusion criteria consisted of the absence of cracks, fractures, anomalies and defects in visual examination and visualization under a stereomicroscope (SMZ1500, Nikon, Tokyo, Japan). The teeth were placed in a 0.5% chloramine-T trihydrate bacteriostatic/bactericidal solution (Merck KGaA, Darmstadt, Germany) for 7 days and then stored in distilled water in a refrigerator at 4ºC, with renewal of the storage medium regularly. At a 24-hour interval before the initiation of the procedural steps of the study, the teeth were transferred into distilled water at 23±2ºC for conditioning.

Cl V cavities were prepared on the buccal surfaces of all the teeth, measuring 3×3 mm occluso-gingivally and mesiodistally and 2 mm in depth. The occlusal wall was adjusted 1.5 mm coronal to the CEJ, with the gingival wall 1.5 mm apical to it. A diamond fissure bur (Diatech Dental AG, Swiss Dental Instruments, CH-9435 Heerbrugg) was used to prepare the cavities in a high-speed handpiece using air and water spray. One new bur was used for every five cavities. No bevels were placed at cavity margins. During the cavity preparation procedures, the tooth surfaces were kept moist to protect them against dehydration. Subsequently, the tooth samples were divided into two groups (n=30) in a random manner. In group 1, BeautiBond (Shofu Inc., Kyoto, Japan) self-etch adhesive was applied based on manufacturer’s instructions, followed by light-curing for 10 seconds with Astralis 7 (Ivoclar Vivadent, Schaan, Liechtenstein) light-curing unit at a light intensity of 400 mW/cm2. The cavities were restored with Beautifil II (Shofu Inc., Kyoto, Japan) giomer incrementally using two one-mm-thick layers. Each layer underwent light-curing for 40 seconds at 400 mW/cm2. One operator carried out all the restorative procedures. Then diamond finishing burs (Diamont Gmbh, D&Z, Berlin, Germany) and polishing disks (Sof-Lex, 3M ESPE, Dental Products, St. Paul, MN, USA) were used to polish all the samples.

Subsequently, the samples were stored in distilled water at 37ºC for 24 hours. To simulate the oral cavity conditions, the samples were thermocycled at 5±2/55±2ºC, which consisted of 500 rounds with 30 seconds of dwell time and 10 seconds for transferring the samples.

In group 2, all the procedural steps were similar to those in group 1 except for the fact that after preparation of the cavity 2% chlorhexidine gluconate disinfecting solution (Consepsis, Ultradent Products, South Jordan, Utah, USA) was used to disinfect the cavity. A minibrush was used to apply 2% CHX solution to the cavity walls, which was left to remain in contact with the cavity walls for 20 seconds, followed by drying for 15 seconds with an air syringe (3).

The tooth samples were finally sectioned buccolingually at the middle of the restorations, using a diamond disk (Diamont Gmbh, D&Z, Berlin, Germany). Then the ×40 magnification of a stereomicroscope (SMZ1500, Nikon, Tokyo, Japan) was used to measure the gap sizes at gingival margins (16). Digital photographs were taken from selected areas using a DS-L2 control unit (Nikon, Tokyo, Japan) in order to measure the gap sizes. The gaps were measured using the built-in software by using a tangential line on the tooth-side vector to determine the distance between the points on the restoration-side vector and the above line. The measurements were repeated at three sites: the outer, middle and inner portions of the gingival margins. The means of marginal gap sizes at these three sites were calculated in micrometers in each study group. Regarding the observer concordance and reproducibility, first ten samples gaps were measured by two observers. Then Intraclass Correlation Coefficient (ICC) was calculated between gap values obtained by two observers. It was about 89%. The ICC was considered excellent when the ICC value was greater than 0.8 (17). Therefore, one observer continued the gap measurement of the rest of the samples.

**Figure 1:**
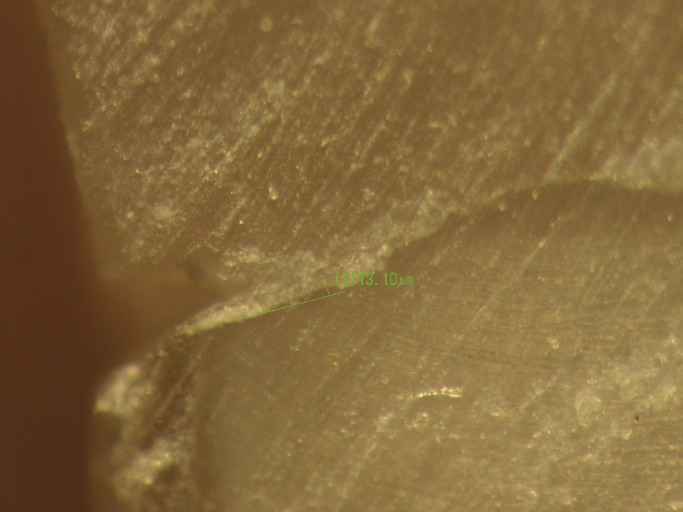
A digital photograph of marginal gap in the study sample.

**Figure 2:**
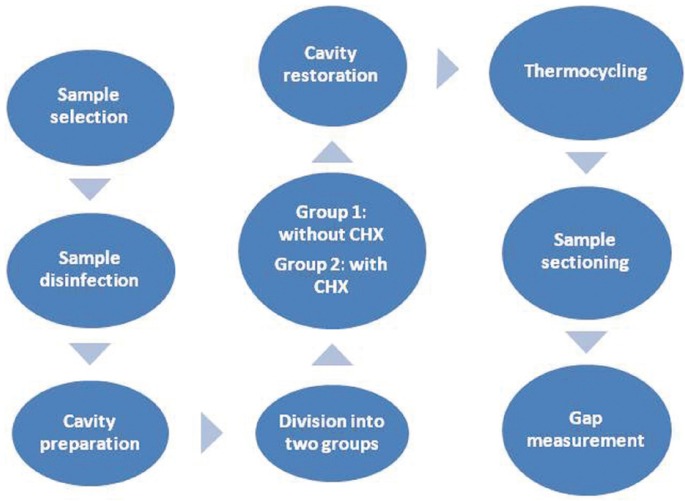
A diagram for sample preparation and the methodology process.

A digital photograph of marginal gap in the study sample has been shown in figure 1. Figure 2 shows a diagram for sample preparation and the methodology process of the present study. Marginal gap data were analyzed in both groups with Mann-Whitney U test because Kolmogorov-Smirnov test showed that distribution of data was not normal (*P*<0.001) and Levene’s test showed that variances were unequal (*P*=0.003). SPSS 20.0 was used for statistical analyses. Statistical significant was defined at *P*<0.05.

## Results

**Figure 3:**
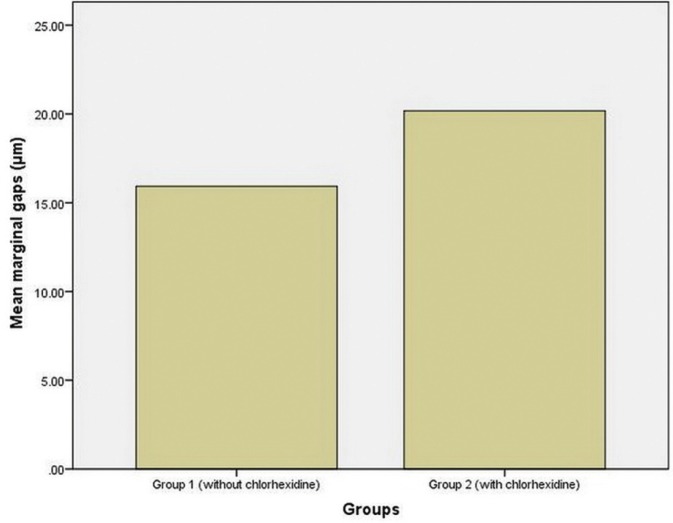
Bar Graph of mean values of marginal gaps (in µm) in the two study groups.

Figure 3 presents the bar graph of mean values of marginal gaps in the two study groups. In groups 1 and 2 the means and standard deviations of marginal gaps were 15.93±2.10 µm and 20.17±4.99 µm, respectively.

The results of Mann-Whitney U test showed significant differences in the means of marginal gaps between the two study groups (U=180, *P*<0.001), with higher means of marginal gaps in group 2 (with CHX disinfection) compared to group 1 (without CHX disinfection) (*P*<0.0005).

## Discussion

In the present study, the effect of a disinfecting agent (2% CHX) on gap formation at gingival margins of giomer restorations was evaluated with the use of a one-step self-etch adhesive. Based on the results, the mean gaps sizes in restorations with the use of CHX for disinfection before the use of the adhesive agent were significantly higher than those in cavities in which CHX was not used.

In this context, Tulunoglu *et al.* evaluated the effect of two disinfecting agents, a CHX-based and an alcohol-based agent, in the cavity on composite resin restorations before the application of two one-bottle total-etch adhesives (Syntac and Prime & Bond) and concluded that use of CHX solution resulted in a significant increase in dentin microleakage in deciduous teeth. However, the alcohol-based disinfecting agent did not have any significant effect on the sealing ability of the above bonding agents (9). In addition, a study showed that use of CHX resulted in a significant increase in microleakage at dentin and enamel margins of composite resin restorations bonded with a one-bottle total-etch adhesive (PQ1) in permanent teeth (18). Meiers and Shook reported a significant decrease in the shear bond strength to dentin with the use of a two-step total-etch bonding agent (Syntac) with the use of CHX before the application of the bonding agent (19). In addition, another study showed that use of CHX before the application of two-step self-etch adhesives (Clearfil SE Bond and Clearfil Protect Bond) decreased immediate bond strength to dentin (20).

The increase in gap formation and the decrease in sealing ability after the use of CHX in giomer restorations bonded with the use of BeautiBond one-step self-etch adhesive might be attributed to the resistance of CHX-treated dentin surfaces to acid conditioning. Based on the classification of self-etch adhesives according to their pH values (21), BeautiBond is considered a mild adhesive with a pH value of 2.4. SEM evaluations have shown that dentin disinfecting agents that are used in the cavity are resistant to acid conditioning (22). In addition, it has been reported that CHX deposits debris on the surface and within the dentinal tubules (23). It appears the acid-resistant layer created due to the effect of CHX might inhibit the demineralizing effect of BeautiBond adhesive on the dentin surface since this adhesive has a high pH value and less acidity. In addition, the remaining debris can decrease the saturation capacity of the dentin surface by resin.

Contrary to the results of the present study, in a study by Silva *et al.* use of CHX did not cause loss of dentin bond strength of two-step etch-and-rinse adhesives (Ambar and Single Bond 2) (12). Pappas *et al.* reported an increase in the shear bond strength of a one-step self-etch adhesive (All Bond 2) to dentin after the application of a 3-step disinfection process (CHX, Tubulicid Red, NaOCl) before the bonding procedure (24). It has been demonstrated that CHX prevents degradation of collagen at the bonded interface over time through inhibition of matrix metalloproteinases (MMPs) (25), resulting in a decrease in the loss of the bond after 6 months of aging (20). Francisconi-dos-Rios *et al.* reported that use of CHX conserved the bond strength of the etch-and-rinse adhesive (Adper Single Bond 2) to both sound and eroded dentin after 6-month aging (13,14). Moreover, Breschi *et al.* concluded that CHX significantly lowered the loss of bond strength and nanoleakage seen in acid-etched resin-bonded dentin which was artificially aged for two years (26). In a study by Carrilho *et al.* it has been suggested that application of CHX might be useful for the preservation of dentin bond strength in etch-and-rinse adhesive (27). In this regard, Ricci *et al.* showed that use of CHX was capable of reducing the rate of resin-dentin bond degradation within the first few months after restoration (28).

It appears use of a cavity disinfecting agent along with restorative adhesive agents yields different results depending on the material used and its interactions and reactions with different bonding systems (1,9,22). The sequence of the application of disinfecting agents depends on the bonding system used. In the total-etch adhesive system since the smear layer and the surrounding dentin are removed, it is rational to apply the disinfecting agent after etching the cavity. However, the self-etch systems with a weaker acidic primer only modify the smear layer and it is necessary to use the disinfecting agent before applying the acidic primer (5). The discrepancies between the results of the above studies (12-14,24,26-28) and the present study might be attributed to differences in the chemical structure of the adhesives used, differences in the methodology (evaluation of the bond strength instead of evaluation of the gap), combination of the disinfecting agent with other irrigation solutions and use of CHX after acid etching step of the total-etch adhesive resin. Considering the multiplicity of factors affecting the oral cavity, it is suggested that *in vivo* studies be carried out to evaluate the performance of different disinfecting agents in the long term in giomer restorations.

Under the limitations of the present study, it can be concluded that use of 2% CHX to disinfect the cavity before application of a one-step self-etch adhesive system with giomer restorations resulted in an increases in gap formation at gingival margins.
